# Using iRNA-seq analysis to predict gene expression regulatory level and activity in *Zea mays* tissues

**DOI:** 10.1093/g3journal/jkac086

**Published:** 2022-04-11

**Authors:** Lauren M Schulte, Kathryn M Koirtyohann, Karen M McGinnis

**Affiliations:** Department of Biological Science, Florida State University, Tallahassee, FL 32306, USA

**Keywords:** transcriptional activity, gene regulation, maize, *Zea mays*, iRNA-seq analysis

## Abstract

Plants regulate gene expression at the transcriptional and post-transcriptional levels to produce a variety of functionally diverse cells and tissues that ensure normal growth, development, and environmental response. Although distinct gene expression patterns have been characterized between different plant tissues, the specific role of transcriptional regulation of tissue-specific expression is not well-characterized in plants. RNA-seq, while widely used to assay for changes in transcript abundance, does not discriminate between differential expression caused by mRNA degradation and active transcription. Recently, the presence of intron sequences in RNA-seq analysis of libraries constructed with total RNA has been found to coincide with genes undergoing active transcription. We have adapted the intron RNA-sequencing analysis to determine genome-wide transcriptional activity in 2 different maize (*Zea mays*) tissues: husk and V2-inner stem tissue. A total of 5,341 genes were predicted to be transcriptionally differentially expressed between the 2 tissues, including many genes expected to have biological activity relevant to the functional and developmental identity of each tissue. Correlations with transcriptional enhancer and transcription factor activity support the validity of intron RNA-sequencing predictions of transcriptional regulation. A subset of transcription factors was further analyzed using gene regulatory network analysis to determine the possible impact of their activation. The predicted regulatory patterns between these genes were used to model a potential gene regulatory network of transcription factors and regulatory targets.

## Introduction 

Gene expression affects the development, physiology, and environmental responsiveness of a plant and is regulated to ensure normal growth and development. Regulation of gene expression can occur at the transcriptional and the post-transcriptional levels to produce a variety of functionally diverse cells and tissues. Transcriptional regulation controls the production of pre-mRNA via mechanisms that include transcription factor (TF) binding, enhancers, and the manipulation of chromatin states. Post-transcriptional regulation often involves changes in mRNA stability and degradation. Collectively, different regulatory mechanisms will determine the abundance and persistence of a specific mRNA across tissues and developmental stages.

RNA-seq is widely used for analyzing gene expression. This approach assays for relative changes in transcript abundance based on the presence or absence of sequencing reads representing exons within a gene model, and therefore does not discriminate between differential expression caused by mRNA degradation and active transcription. In eukaryotes, most protein-coding genes are transcribed by RNA polymerase II (Pol II) into pre-mRNA, which are nascent RNA molecules that include both introns and exons. Post-transcriptional processing of a pre-mRNA results in a mature mRNA that has been spliced to only include exons and includes a poly(A) tail and 5′-cap. The level of post-transcriptional regulation that impacts steady-state transcript levels vary for different gene products. Recently, it was discovered that the presence of intronic sequence reads in RNA-seq analysis of total cellular RNAs corresponds with genes undergoing active transcription (Gaidatzis *et al.* 2015), while other approaches to analyzing RNA-seq data assay expression based on exons only. This observation forms the basis of the intron RNA-sequencing (iRNA-seq) analysis pipeline, which assesses intron coverage as a surrogate for active transcriptional and post-transcriptional changes in total RNA-rRNA depleted sequencing datasets to identify changes in gene expression (Madsen *et al.* 2015).

Differences in the regulation of gene expression can vary between genes within gene families (Teichmann and Babu 2004; Atkinson and Halfon 2014; Zhong *et al.* 2019). Genome duplication produces and expands gene families, which are sets of similar genes formed by the duplication of an original gene. Duplicated genes can often evolve different expression patterns related to a variation in function. These different expression patterns can be due to different promoter elements, which can influence gene expression across tissues or cell types, giving rise to the various gene functions (Guschanski *et al.* 2017; Zhong *et al.* 2019).

In modern maize (*Zea mays*), the genome structure has been altered by several genome duplication events. The first major event occurred approximately 70 million years ago in a cereal ancestor (Paterson *et al.* 2004). Another genome duplication occurred 11.9 million years ago, and this duplication is estimated to have occurred at the same time as the divergence between maize and its closest ancestor, *Sorghum bicolor* (Blanc and Wolfe 2004; Swigonová *et al.* 2004). Two subgenomes of maize were identified because of this duplication and have been designated as Maize1 and Maize2 (Schnable *et al.* 2011). These 2 subgenomes are distinguished by the expression of retained duplicate genes and the rate of gene loss. Maize1 contains a greater proportion of genes orthologous to rice and sorghum, and thus the tetraploid ancestor of maize, compared to Maize2 (Schnable *et al.* 2011). Maize1 also has experienced less gene loss and Maize1 genes are typically found to be expressed at higher levels in modern maize (Schnable and Freeling 2011). In addition to general differences in level of expression, there can be tissue- and developmental-stage-specific changes in expression of duplicate genes.

Here, we have adapted the iRNA-seq analysis pipeline to evaluate transcriptional activity genome-wide in 2 different tissues of maize, B73 husk tissue and B73 V2-inner stem tissue (IST), by utilizing a previously published total RNA-rRNA depleted sequencing dataset (GSE94252; Oka *et al.* 2017). We demonstrate that iRNA-seq analysis is an effective way to predict the relative contribution of transcriptional changes to gene expression and regulation in these 2 tissues, thus improving our understanding of developmental gene regulation in maize.

## Methods

### Adding the maize genome to the iRNA-seq pipeline

The maize genome annotation gff3 file (Ensembl AGPv4 release 38 genome; contigs included; Kersey *et al.* 2018) was converted using the gff3ToGenePred tool in the Blat program suite (Kent 2002) to GenePred format. Minor edits to column format were made in the command line to produce a “refGene” table that matched the tables available in the UCSC Genome Browser.

In addition, a Blat v.36x2 (Kent 2002) was performed between the maize genome fasta file (Ensembl AGPv4 release 38 genome; contigs included; Kersey *et al.* 2018) and a file containing all available maize mRNA sequences extracted from the NCBI GenBank (Included 171,280 sequences) to produce an “all_mRNA” table in psl format. The “all_mRNA” table was further refined by the pslReps tool, included in the Blat program suite (Kent 2002). Minor edits to column format were made in the command line to produce an “all_mRNA” table that matched the tables available in the UCSC Genome Browser.

The “refGene” and “all_mRNA” tables were renamed “Gene.Dump” and “mRNA.Dump,” respectively, and added into the “tmp” folder within the iRNA-seq pipeline.

The “Analyze.R” script, included in the iRNA-seq pipeline, was modified to function with the maize gene ID length (gene ID nomenclature is often unique to a genome).

The “AddGenome.sh” script, included in the iRNA-seq pipeline (Madsen *et al.* 2015), was modified to include the genome designation (AGPv4_ctg) and used to create the maize exon and intron lists needed to perform iRNA-seq analysis. These exon and intron lists do not include any overlapping regions, thus taking into consideration any possible differences due to alternative splicing. The complete iRNA-seq pipeline is available on GitHub (https://github.com/lmschulte/iRNA-seq).

### Total RNA-rRNA depleted extraction, library preparation, sequencing, and processing

Total RNA-rRNA depleted-seq data from B73 husk tissue and B73 V2-IST was previously generated (Oka *et al.* 2017; GSE94252). As described previously (Oka *et al.* 2017), in brief, RNA was extracted with TRIzol (ThermoScientific) with some modification to manufacturer’s instructions and further processed by the RNeasy kit (Qiagen). Next, samples were treated with DNase I (DNA-free kit, Ambion) according to the manufacturer’s instructions to remove remanent DNA. Samples were extracted with 1 volume of phenol: chloroform: isoamyl alcohol (25:24:1 v/v) and centrifuged. The same step was repeated twice. Next, 80% of the aqueous phase volume was transferred into a new tube and precipitated with 1/10th volume of 3 M Sodium Acetate pH 5.6, 2 volumes of 100% ethanol and 1 μl of glycogen (10 mg/ml), followed by centrifugation. The pellet was subsequently washed twice with 70% ethanol and finally resuspended in RNase-free water. The concentration was measured with a Nanodrop spectrophotometer (ThermoScientific) and 1 μg of RNA was separated on a 1.2% agarose 1× MOPS (3-N-morpholinol propane sulfonic acid) gel to assess RNA quality. Ribosomal RNA was removed from 500 ng of total RNA using the Ribo-Zero rRNA Removal Kit (Plant Leaf, Epicentre). RNA-seq libraries were prepared with the NEBNext Ultra Directional RNA Library Prep Kit for Illumina sequencing (New England Biolabs). Quality and quantity were assessed at all steps of the library preparation by capillary electrophoresis (Agilent Bioanalyzer and Agilent Tapestation). Single-end 100 bp sequencing was performed with TruSeq v3 chemistry on a HiSeq2500. Approximately 15–20 million reads were obtained for each library, except for 1 sample (Table 1). The total RNA-rRNA depleted-seq library data was downloaded as SRA files from the NCBI gene expression omnibus (GEO) with 6 biological replicates per tissue type (GSE94252). SRA files were converted to fastq files using the SRA Toolkit 2.9.6-1 (http://ncbi.github.io/sra-tools/). RNA-seq reads were aligned to the maize genome (Ensembl AGPv4 release 38; contigs included) index created with STAR 2.6 (Dobin *et al.* 2013) using default STAR parameters.

**Table 1.** Summary of RNA-seq libraries for IST and husk tissue.

Tissue	Replicate	**Sample name** ^*a*^	Total raw reads	Mapped reads	% Mapped reads	Uniquely mapped reads	% Uniquely mapped reads
Husk	1	Husk.rep1.MPI	18,019,379	17,889,371	99.28	17,069,922	94.73
Husk	2	Husk.rep1.UvA	1,730,157	1,716,575	99.21	1,641,331	94.87
Husk	3	Husk.rep2.MPI	17,513,029	17,385,239	99.27	16,608,229	94.83
Husk	4	Husk.rep2.UvA	18,692,557	18,559,542	99.29	17,769,401	95.06
Husk	5	Husk.rep3.MPI	16,368,453	16,252,861	99.29	15,520,416	94.82
Husk	6	Husk.rep3.UvA	14,895,265	14,776,948	99.21	14,115,411	94.76
IST	1	IST.rep1.MPI	16,626,209	16,420,079	98.76	15,023,528	90.36
IST	2	IST.rep1.UvA	15,881,040	15,775,278	99.33	15,018,425	94.57
IST	3	IST.rep2.MPI	15,830,192	15,594,675	98.51	14,094,774	89.04
IST	4	IST.rep2.UvA	16,260,713	16,147,099	99.30	15,368,002	94.51
IST	5	IST.rep3.MPI	14,684,139	14,506,790	98.79	13,287,765	90.49
IST	6	IST.rep3.UvA	15,625,655	15,477,981	99.05	14,748,193	94.38

a Included 100-bp single-end reads (GSE94252; Oka *et al.* 2017).

### iRNA-seq analysis

iRNA-seq analysis requires libraries that have not been fractionated by poly-A selection, because this type of library would be enriched for fully processed mature mRNAs and would lack the nascent pre-mRNA transcripts mostly like to contain introns. Suitable publicly available datasets were identified for this study, including total RNA-rRNA depleted seq data obtained from B73 husk and V2-IST in maize (GSE94252; Oka *et al.* 2017). iRNA-seq analysis of the processed total RNA-rRNA depleted-seq data from B73 husk tissue and B73 V2-IST was performed using the iRNA-seq pipeline to count introns and exons, producing 2 output files. The output files were first filtered for an adjusted *P*-value (*P*adj) < 0.05, and then genes with a Log2_FC > 1 were determined as husk and genes with a Log2_FC < −1 were determined as IST. Genes were further sorted as being intron only, exon only, or containing both introns and exons. Genes with introns only and both introns and exons were predicted to be transcriptionally differentially expressed, while genes only containing exons were predicted to be post-transcriptionally differentially expressed (Supplementary Files 1–4). In addition, genes predicted to be post-transcriptionally differentially expressed were further sorted to only include genes with multiple exons annotated in the gene model, as these genes have the potential to be predicted as transcriptionally differentially expressed and comparable (Supplementary Files 3 and 4). Genes predicted as multiple exon post-transcriptionally differentially expressed were used in all future analyses involving post-transcriptionally differentially expressed genes. The complete iRNA-seq analysis pipeline is available on GitHub (https://github.com/lmschulte/iRNA-seq).

### Plant materials

Maize (*Z.* *mays*) plants of the B73 background were grown in a greenhouse until they reached the V2 stage. IST was harvested, and flash frozen in liquid nitrogen and stored at −80°C until use. IST tissue was used for reverse transcription quantitative PCR (RT-qPCR) analysis.

### Nuclear and cytoplasmic RNA extractions

Frozen IST tissue was finely ground into powder in liquid nitrogen and homogenized. To isolate nuclear and cytoplasmic RNA, nuclei were isolated from 1 g of IST tissue as described previously (Stroud and McGinnis 2017) with several modifications. Frozen, ground IST tissue was added to ice-cold modified Apel buffer (20 mM Tris‐HCl pH 7.8, 250 mM sucrose, 5 mM MgCl_2_, 5 mM KCl) and 0.1 M phenylmethylsulfonyl fluoride by gently stirring for 10 min on ice. While stirring, 2.5 M glycine was added for 5 min. The cells were lysed by continuing to stir on ice for 5 min with 0.25% Triton X‐100 (EMD Millipore) and 0.1% β-mercaptoethanol. This mixture was then filtered through 2 layers of Miracloth (EMD Millipore). Filtered samples were then centrifuged at 14,000 g for 10 min at room temperature in a Sorvall RC 6 Plus centrifuge (with accelerate at 9 and decelerate at 9). Nuclei were isolated in the pellet with cytoplasm in the supernatant. The nuclear pellet was resuspended in 1 ml of MNase digestion buffer and the supernatant cytoplasm samples were directly aliquoted, then all samples were flash frozen in liquid nitrogen and stored at −80°C until use. Nuclear and cytoplasm RNA was extracted from each sample using TRI Reagent according to the manufacturer’s instructions (Molecular Research Center, TR 118). RNA samples were DNase treated (RQ1 RNase-free DNase, Promega, M6101) and purified using the Zymo Research RNA Clean & Concentrator -25 Kit (R1017). The RNA quality and quantity was measured by a NanoDrop 2000c spectrophotometer.

### Reverse transcription quantitative PCR (RT-qPCR) analysis

RT-qPCR was performed for 3 nuclear RNA samples and 3 cytoplasmic RNA samples to confirm iRNA-seq analysis results in IST. The *U6* gene (Zm00001d017432) was used as a nuclear RNA control, since U6 snRNA is only present in the nucleus, and *Ubiquitin conjugase* (Zm00001eb203340) was used a cytoplasmic RNA control, as it is a highly expressed gene across tissues. Differentially expressed genes identified by iRNA-seq analysis were randomly selected. Selected genes and primer information can be found in more detail in Supplementary files (Supplementary File 14).

First-strand cDNA synthesis and RT-qPCR were performed by Florida State University’s Biology Molecular Core Facility. First-strand cDNA synthesis was performed by reverse transcribing 160 ng of total RNA with random hexamers according to manufacturer’s instructions (SuperScript III Reverse Transcriptase, Invitrogen, 18080-051). Reverse transcriptase quantitative PCR (RT-qPCR) was performed using an Applied Biosystems Quantstudio 7 Flex and SYBR Green reagents (Quantabio). The generation of specific PCR products was confirmed by melting curve analysis. Primers were designed using NCBI Primer BLAST (https://www.ncbi.nlm.nih.gov/tools/primer-blast/index.cgi) and Maize GDB (https://www.maizegdb.org/).

### Gene ontology

Gene ontologies for biological process (P), molecular function (F), and cellular component (C) were determined for genes transcriptionally differentially expressed (2,573 husk genes and 2,768 IST genes) using the online tool, agriGO v2.0 (Tian *et al.* 2017). The singular enrichment analysis (SEA) tool was selected and used with the following parameters: maize v4 (Maize-GAMER) reference genome, the Fisher statistical method, the Yekutieli (FDR under dependency) multitest adjustment method, the Plant gene ontology (GO) Slim GO type, 0.01 significance level, and a minimum of 10 mapping entries. The GO term enrichment heatmap was generated by hierarchically clustering the log10 of the total GO term percentage of a set of genes that were transcriptionally differentially expressed in either IST or husk tissue (Supplementary File 5).

### Enhancer analysis

In a previous study, lists of tissue-specific genes linked to tissue-specific enhancers were identified for IST and husk tissue (Oka *et al.* 2017, Supplementary File 5). Any genes with only 1 exon, and thus no introns, were removed. These modified lists were compared to the iRNA-seq transcriptionally (5,341 genes) and multiple exons post-transcriptionally differentially expressed genes (2,747 genes). The percentage of tissue-specific genes linked to tissue-specific enhancers transcriptionally and post-transcriptionally differentially expressed were then determined (Supplementary Files 6 and 7).

### TF analysis

The GO for molecular function (F) conducted by agriGO v2.0 (Tian *et al.* 2017) identified transcriptionally differentially expressed genes in IST (133 genes) and husk tissue (202 genes) with “TF activity or sequence-specific DNA binding activity” (GO: 0003700). These 2 lists of predicted TFs were converted into maize version 3 gene IDs (B73 AGPv3 genome; 5 b+) using a Maize GDB gene ID translation tool (Portwood *et al.* 2019) and processed by the Maize Tissue gene regulatory network (GRN; https://www.bio.fsu.edu/mcginnislab/mgrn/; Huang *et al.* 2018) to identify potential TF target genes (Supplementary Files 8 and 9). The Maize Tissue GRN was designed to only use the AGPv3 genome, so the gene IDs must be translated before use. All available tissues (leaf, root, SAM, and seed) and the TSV file with all information were selected for each TF. The resulting output gave predicted TF target genes for the various TFs organized by tissue and included gene details. The predicted target genes were then compared to the genes identified by the iRNA-seq pipeline as transcriptionally differentially expressed (5,341 genes) and multiple exons post-transcriptionally differentially expressed (2,747 genes). The VennPlex tool (Cai *et al.* 2013) was used to easily compare and sort the various gene lists by transcriptional activity (Supplementary Files 8 and 9).

### Subgenome analysis

In a previous study, 2 maize subgenomes, Maize1 and Maize2, were identified (Schnable *et al.* 2011; updated in Zhang *et al.* 2017). Genes identified by iRNA-seq as transcriptionally differentially expressed (5,341 genes) and multiple exons post-transcriptionally differentially expressed (2,747 genes) were compared to the maize subgenomes to determine subgenome identity (Supplementary File 10).

### Gene family analysis

The gene families of differentially expressed genes were identified using an online tool called GenFam (Bedre and Mandadi 2019; https://www.mandadilab.com/genfam/). To use the GenFam gene IDs had to be converted into the maize version 3 gene ID (B73 AGPv3 genome; 5 b+) using the Maize GDB gene ID translation tool (Portwood *et al.* 2019). GenFam follows the Phytozome v12.0 database format, which uses the AGPv3 genome, so the gene IDs must be translated before use. Default parameters were used, and significant families have a *P*-value < 0.05.

### TF hierarchical regulatory network

Promoter sequences of the genes comprising the TUBBY-like (TLP) and Alfin-like (AL) gene families were taken as the 2,500 bp before the first exon as determined on Ensembl Plants (Bolser *et al.* 2017). These promoter sequences were then analyzed using PlantPAN 3.0 (Chow *et al.* 2019). Default parameters were used for the multiple promoter analysis.

TLP and AL TFs were converted into maize version 3 gene IDs (B73 AGPv3 genome; 5 b+) using the Maize GDB gene ID translation tool (Portwood *et al.* 2019) and were processed with the Maize Tissue GRN (https://www.bio.fsu.edu/mcginnislab/mgrn/; Huang *et al.* 2018) to identify potential regulatory targets. The Maize Tissue GRN was designed to only use the AGPv3 genome, so the gene IDs must be translated before use. All available tissues (leaf, root, SAM, and seed) and the TSV file with all information were selected for each TLP and AL TF. The resulting output gave predicted TF target genes for the various TFs organized by tissue and included gene details. These targets were compared to genes transcriptionally (5,341 genes) and multiple exon post-transcriptionally differentially expressed (2,747 genes) between the gene families to determine tissue-specific regulation and pathways (Supplementary File 12).

GO for biological process was determined for all the TLP and AL regulatory targets transcriptionally differentially expressed (5,341 genes) using the online tool, agriGO v2.0 (Tian *et al.* 2017). The SEA tool was selected and used with the following parameters: maize v4 (Maize-GAMER) reference genome, the Fisher statistical method, the Yekutieli (FDR under dependency) multitest adjustment method, the Plant GO Slim GO type, 0.01 significance level, and a minimum of 10 mapping entries. The GO output is organized by TLP and AL gene name (Supplementary File 13).

## Results

### iRNA-seq analysis identifies differential transcription in husk and IST

To investigate the relative contributions of transcriptional and post-transcriptional regulation to gene expression in maize, a computational method called iRNA-seq analysis (Madsen *et al.* 2015) was adapted for use with maize datasets and performed on total RNA-rRNA depleted seq data obtained from B73 husk and V2-IST in maize (GSE94252; Oka *et al.* 2017). A total of 10,358 genes were identified as differentially expressed between husk and IST (Table 2), meaning that the transcript level for these genes were higher in 1 tissue compared to the other at the established thresholds. Of these genes, approximately 48% only had exon coverage, 9% only had intron coverage, and 43% had both exon and intron coverage, which enabled predictions of the level of regulation leading to differential transcript abundance. Differentially expressed genes with intron representation were predicted to be pre-mRNAs, and thus indicative of a gene undergoing acute transcriptional activity. Differentially expressed genes with only exon representation were predicted to be mature mRNAs, suggesting that differential expression was related mainly to post-transcriptional mechanisms. A total of 5,341 genes were predicted to be transcriptionally differentially expressed, with 2,573 genes in husk tissue (Supplementary File 1) and 2,768 genes in IST (Supplementary File 2), and 5,020 genes were predicted to be post-transcriptionally differentially expressed, with 2,663 genes in husk tissue (Supplementary File 3) and 2,357 genes in IST (Supplementary File 4). These post-transcriptionally differentially expressed genes were further sorted to only include genes that had multiple exons, as iRNA-seq analysis can only accurately categorize genes with at least 1 intron, which requires 2 or more exons. After this additional sorting, a total of 2,747 genes were predicted to be post-transcriptionally differentially expressed, with 1,368 genes in husk tissue and 1,379 genes in IST (Table 2; Supplementary Files 3 and 4). Only the post-transcriptionally differentially expressed genes with multiple exons were used in further analyses. Additionally, 3 genes were predicted to be transcriptionally differentially expressed in 1 tissue, but post-transcriptionally differentially expressed in the other leading to these 3 genes being included in multiple categories. To test the iRNA predictions, a subset of genes were selected for qRT-PCR analysis of mRNA abundance in nuclear and cytoplasmic fractions of RNA from IST. Although some of the differences were subtle and not statistically significant, mRNAs for genes predicted to be actively transcribed in IST by iRNA-seq were found to be enriched in the nuclear fraction of RNAs extracted from IST (Supplementary Fig. 1, a–c), and 1 gene of 2 genes that were predicted to be transcriptionally active in husk were not enriched in the nuclear fraction of IST (Supplementary Fig. 1e). Notably, we were able to detect a slight enrichment in the nuclear fraction for at least 1 gene when primers were used that spanned intron-exon junctions (Supplementary Fig. 1, a, b, d, and e) or were completely within an intron (Supplementary Fig. 1c).

**Table 2.** Transcriptional activity in IST and husk tissue determined by iRNA-seq analysis.

Transcriptional activity^*a*^	Husk	IST	Total^*d*^
Transcriptionally differentially expressed genes	2,573	2,768	5,341
Post-transcriptionally differentially expressed genes^*b*^	1,368	1,379	2,747
Inconclusive differentially expressed genes^*c*^	1,295	978	2,273

a Differential transcriptional activity was predicted by iRNA-seq analysis and classified as any genes with *P* < 0.05, log2_FC > 1 for husk genes, and log2_FC < −1 for IST genes (Supplementary Files 1–4).

b Post-transcriptionally differentially expressed genes identified by iRNA-seq analysis with multiple exons (Supplementary Files 3 and 4).

c Genes were originally identified as post-transcriptionally differentially expressed by iRNA-seq analysis, but they only contain 1 exon and no introns so it is inconclusive if they are undergoing active transcription and will not be used in further analysis.

d Three genes were included in multiple categories (Supplementary Files 1–4).

### Biological function of transcriptionally differentially expressed genes in husk and IST suggests transcriptional regulation is important in tissue identity and function

**Fig. 1.**
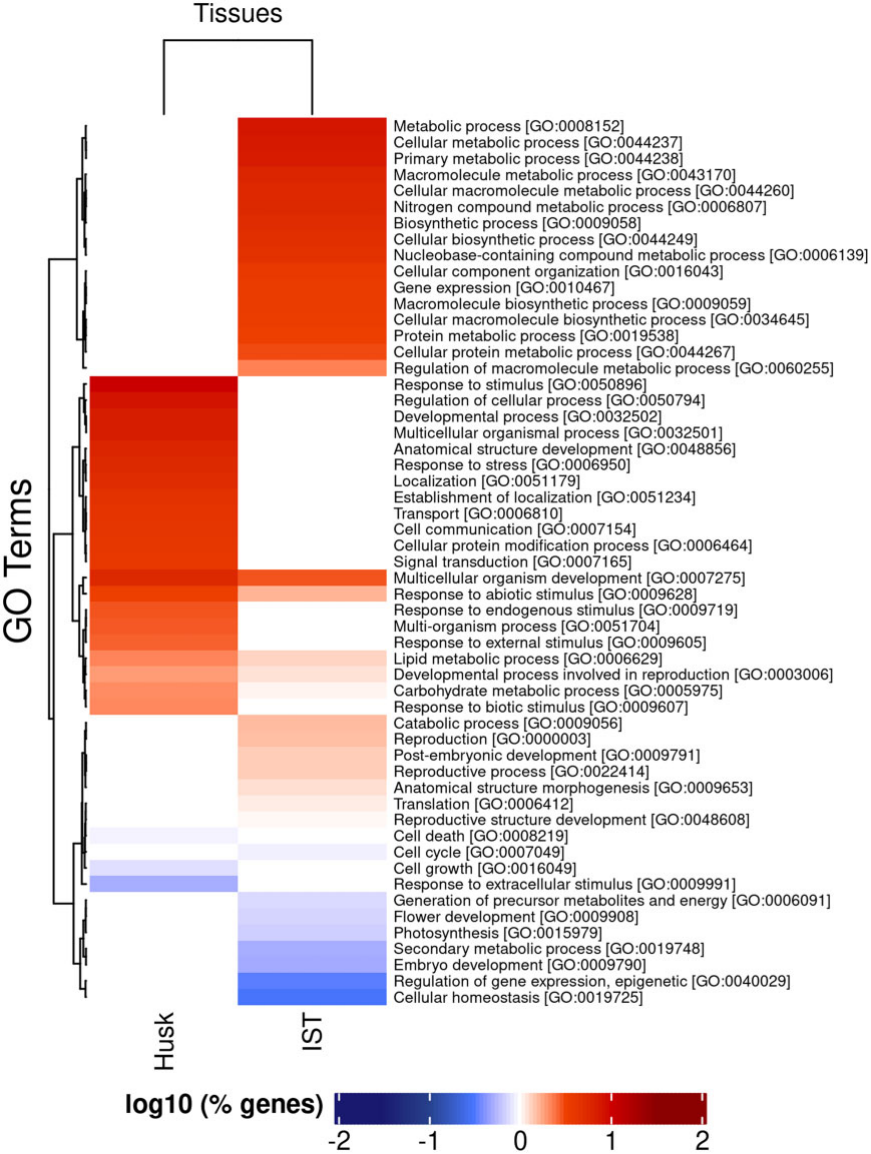
Enriched biological processes GO terms found in transcriptionally differentially expressed genes in husk and IST. Hierarchical clustering of log_10_ (% genes) of significant GO terms enriched in each tissue (FDR < 0.01; *P*-value <0.05; minimum of 10 mapping entries; Supplementary File 5). GO terms and GO accession numbers are labeled for each row.

To determine how transcriptional and post-transcriptional regulation of gene expression may contribute to tissue-specific biological function, genes predicted as transcriptionally differentially expressed were categorized based on a GO analysis of biological function using agriGO v2.0 (Tian *et al.* 2017). There were 25 significant GO terms represented by husk tissue transcriptionally differentially expressed genes and 36 significant GO terms represented by IST transcriptionally differentially expressed genes (Fig. 1; Supplementary File 5). For the genes with increased transcriptional activity in husk tissue, the most common GO terms included “response to stimulus,” “regulation of cellular process,” “developmental process,” “multicellular organismal process,” and “anatomical structure development” (Fig. 1), which are genes that may support the biological function of the photosynthetically active husk tissue. Metabolic processes GO terms were the most common for genes with increased transcription in IST, but GO terms for genes related to reproductive development were also included (Fig. 1), which could be representative of the inclusion of undifferentiated shoot meristem tissues poised to undergo development/differentiation in the sample. Additionally, IST-transcribed genes also included GO terms involved with gene expression, epigenetics, and translation (Fig. 1), which is consistent with actively growing cells and tissues during early vegetative development.

### Predicted enhancer activity corresponds with transcriptional activity in IST and husk tissue

Enhancers mediate a dynamic form of transcriptional regulation and often act in a tissue-specific manner, meaning that tissue-specific enhancer activity would likely coincide with tissue-specific active transcription. In the original study that generated the dataset used herein, tissue-specific genes with candidate enhancers were identified in IST and husk tissue (Oka *et al.* 2017). An increase in IST-specific transcription was identified by iRNA-seq analysis for 80.6% of the genes previously found to be associated with tissue-specific enhancers (Table 3; Supplementary File 6). An increase in husk-specific transcription was identified by iRNA-seq analysis for 79.6% of the genes previously found to be associated with tissue-specific enhancers (Table 3; Supplementary File 7). Biologically, the association of tissue-specific enhancers with tissue-specific transcription is expected, and the overlap between these analyses supports the idea that iRNA-seq analysis is a robust way to identify transcriptional changes in maize.

**Table 3.** Transcriptional and post-transcriptional regulatory activity predicted by iRNA-seq analysis in IST and husk tissue.

	IST	Husk
Tissue-specific genes linked to tissue-specific enhancers^*a*^	31	225
Transcriptionally differentially expressed genes predicted by iRNA-seq^*b*^	25 (80.6%)	179 (79.6%)
Multiple exon post-transcriptionally differentially expressed genes predicted by iRNA-seq^*c*^	6 (19.4%)	46 (20.4%)

a Tissue-specific genes linked to tissue-specific enhancers were defined in previous study (Oka *et al.* 2017) and used for comparison. Those not identified by iRNA-seq analysis were not included.

b The number of tissue-specific genes linked to tissue-specific enhancers that are transcriptionally differentially expressed in either IST or husk tissue as predicted by iRNA-seq, represented as a percentage in parentheses.

c The number of tissue-specific genes linked to tissue-specific enhancers that are multiple exon post-transcriptionally differentially expressed in either IST or husk tissue as predicted by iRNA-seq, represented as a percentage in parentheses.

### TF activity appears to regulate transcriptional activity in IST and husk tissue

**Fig. 2.**
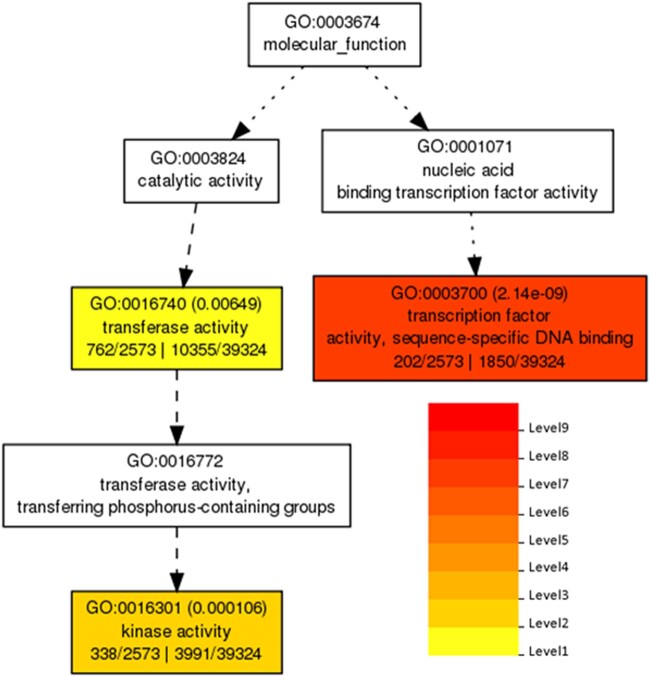
Molecular function GO for transcriptionally differentially expressed husk genes determined using agriGO v2.0 (Tian *et al.* 2017; FDR < 0.01; *P*-value < 0.05; minimum of 10 mapping entries; Supplementary File 5). Color denotes the level of significance with a higher level being more significant

TFs regulate transcription; consequently, genes demonstrating transcriptional activation in IST and husk tissue might be regulatory targets of transcriptionally active TFs in these tissues. Active TFs were identified as transcriptionally differentially expressed genes by iRNA-seq and having the GO term for “TF activity or sequence-specific DNA-binding activity” (GO:0003700). A total of 133 putative TFs were identified as having increases in transcription in IST, and 202 putative TFs were identified as having increases in transcription in husk tissue (Fig. 2). To identify potential regulatory targets of these TFs, they were used to query the Maize Tissue GRN (https://www.bio.fsu.edu/mcginnislab/mgrn/; Huang *et al.* 2018). Although the Maize Tissue GRN does not include these specific tissues and may not be able to predict all potential targets of these TFs, it includes many datasets and allows for the prediction of at least a subset of regulatory targets predicted for these TFs. Of the putative TFs identified, 83 active TFs from IST and 157 active TFs from husk tissue were available for analysis in the Maize Tissue GRN (Table 4; Supplementary Files 8 and 9). The predicted regulatory effect of these TFs on regulatory targets correlates with a substantial subset of transcriptional differences between husk and IST; 92.3% of the genes identified as transcriptionally differentially expressed in IST and 89.9% of the genes identified as transcriptionally differentially expressed in husk tissue were predicted regulatory targets of ‘active’ TFs in the respective tissues. In comparison, of the genes identified as post-transcriptionally differentially expressed, 85.9% in IST and 78.1% in husk tissue were predicted to be TF targets (Table 4; Supplementary Files 8 and 9). These results suggest that TF activity can be correlated with some examples of active transcription within this dataset and in these tissues.

**Table 4.** Transcriptional activity related to transcription factors in IST and husk tissue.

	IST	Husk
Transcription factors^*a*^	83	157
Predicted transcription factor targets identified as differentially expressed by iRNA-seq^*b*^	3,739	3,382
Transcription factor targets identified by iRNA-seq as transcriptionally differentially expressed^*c*^	2,554	2,314
Percentage of transcriptionally differentially expressed genes that are transcription factor targets^*d*^	92.3%	89.9%
Transcription factor targets identified by iRNA-seq as multiple exon post-transcriptionally differentially expressed^*e*^	1,185	1,068
Percentage of multiple exon post-transcriptionally differentially expressed genes that are transcription factor targets^*f*^	85.9%	78.1%

a Transcription factors predicted as transcriptionally differentially expressed by iRNA-seq and defined by inclusion in GO:0003700 in agriGO v2.0 and Maize Tissue GRN (Supplementary Files 8 and 9).

b Predicted transcription factor targets that were identified as differentially expressed by the iRNA-seq pipeline. The total number of predicted targets identified by the Maize Tissue GRN when all tissues and TSV file with all information are selected were 25,172 for IST transcription factors and 26,469 for husk transcription factors (Supplementary Files 8 and 9).

c Predicted transcription factor targets that are transcriptionally differentially expressed in either IST or husk tissue as predicted by iRNA-seq; of which, 2,768 genes were in IST and 2,573 genes were in husk tissue (Supplementary Files 8 and 9).

d The percentage of genes that are transcriptionally differentially expressed, as predicted by iRNA-seq analysis, and are predicted transcription factor targets.

e Predicted transcription factor targets that are multiple exon post-transcriptionally differentially expressed in either IST or husk tissue as predicted by iRNA-seq; of which, 1,379 genes were in IST and 1,368 genes were in husk tissue (Supplementary Files 8 and 9).

f The percentage of genes that are multiple exon post-transcriptionally differentially expressed, as predicted by iRNA-seq analysis, and are predicted transcription factor targets.

### More genes are transcriptionally differentially expressed from Maize1 than from Maize2

The evolutionary history of the maize genome includes a tetraploidy associated with 2 subgenomes, commonly referred to as Maize1 and Maize2 (Schnable *et al.* 2011). In genomes with such duplications, there are frequently fractionation events that lead to the loss of 1 copy of a gene (Langham *et al.* 2004; Schnable *et al.* 2011; Brohammer *et al.* 2018). Genes that are retained as duplicates might also be released from some level of selective pressure, allowing specialization of 1 gene that might include limitation of expression to a subset of tissues. In maize, genes included in Maize1 are typically expressed at a higher level (Schnable and Freeling 2011). To determine if transcriptional activity in tissues is correlated with duplication-associated specialization, genes with differential expression in husk and IST were classified by their subgenome assignments. Overall, Maize1 genes were overrepresented for both transcriptionally and post-transcriptionally differentially expressed genes within IST and husk tissue. When comparing the subgenomes, 54.99% and 55.67% of the genes being transcriptionally differentially expressed in husk tissue and IST identified as Maize1, and 29.89% and 32.26% of the genes being transcriptionally differentially expressed in husk and IST identified as Maize2 (Table 5; Supplementary File 10). A detectable difference in representation exists even if these values are normalized to account for the ∼1.6x larger size of the Maize1 subgenome, which includes 15,146 genes compared to the 9,476 genes in Maize2. The post-transcriptionally differentially expressed genes had similar trends in subgenome identification (Table 5; Supplementary File 10). These results suggest that genes exhibiting transcriptional activity are more likely to belong to the Maize1 subgenome and that Maize1 genes are more transcriptionally active and more likely to be differentially expressed than Maize2 genes in IST and husk tissue.

**Table 5.** Subgenome identity of genes undergoing different levels of transcriptional activity.

Transcriptional activity	Husk			IST		
	Maize 1	Maize 2	No subgenome	Maize 1	Maize 2	No subgenome
Transcriptionally differentially expressed^*a*^	1,415 (54.99%)	769 (29.89%)	389 (15.12%)	1,541 (55.67%)	893 (32.26%)	334 (12.07%)
Post-transcriptionally differentially expressed^*b*^	638 (46.64%)	393 (28.73%)	337 (24.63%)	727 (52.72%)	420 (30.46%)	232 (16.82%)

a The number of transcriptionally differentially expressed genes in either IST or husk tissue as predicted by iRNA-seq in the subgenome, represented as a percentage in parentheses (Supplementary File 10).

b The number of multiple exon post-transcriptionally differentially expressed genes in either IST or husk tissue as predicted by iRNA-seq in the subgenome, represented as a percentage in parentheses (Supplementary File 10).

### Early regulation in TLP and AL gene families illustrates differences in gene expression between these 2 tissues

Gene duplication events can result in groups of highly similar genes that can be identified as gene families. The genes comprising a gene family do not always exhibit the same expression pattern, creating the potential for functional specification and distinct expression patterns between genes with highly similar sequences. To further understand tissue-specific gene expression and the diversification of gene families, we investigated the regulatory mechanisms of gene families that were found for genes that were transcriptionally differentially expressed in both IST and husk tissue (Supplementary File 11). Of the 870 gene families identified, 24 gene families were shared between transcriptionally differentially expressed genes in IST and husk tissue. From this list of shared gene families, we chose to focus on the TLP and AL gene families, because each of these gene families included 1 or more transcriptionally differentially expressed genes in each of the 2 tissues examined. In the TLP family, there was 1 gene that was transcriptionally differentially expressed in each tissue, while in the AL family, 2 genes were transcriptionally differentially expressed in IST and 1 in husk tissue (Table 6). In addition, these 2 gene families are known to act as TFs, giving them the potential to influence gene expression and regulation in these 2 tissues.

**Table 6.** AL and TLP genes transcriptionally differentially expressed in IST and husk tissue.

Gene family	Gene ID^*a*^	Gene name	Gene symbol	Tissue^*b*^	Subgenome^*c*^
AL	Zm00001d037537, GRMZM2G148810	AL-transcription factor 1	alf1	Husk	Maize1
	Zm00001d041767, GRMZM2G153087	AL-transcription factor 4	alf4	IST	Maize 2
	Zm00001d049007, GRMZM2G008259	AL-transcription factor 18	alf18	IST	No subgenome
TLP	Zm00001d038691, GRMZM2G435445	TUB-transcription factor 5	tubtf5	Husk	Maize 1
	Zm00001d015485, GRMZM2G472945	TUB-transcription factor 8	tubtf8	IST	Maize 1

a B73 AGPv4 and AGPv3 genome (also known as 5 b+) gene IDs.

b The tissue identity of the transcriptionally differentially expressed gene according to iRNA-seq analysis.

c The subgenome identity of the gene (Schnable *et al.* 2011; updated in Zhang *et al.* 2017; Supplementary File 10).

**Fig. 3.**
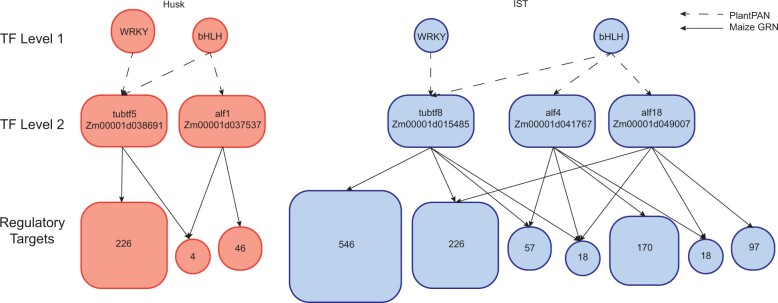
Predicted hierarchical regulatory network for the TLP and AL gene families. Transcription factors in TF Level 1 were predicted through PlantPAN to interact with TLP and AL gene promoters (denoted with dotted line arrows). Regulatory targets of TLP and AL genes were predicted using the Maize Tissue GRN (denoted with solid line arrows) with the number of predicted targets depicted. All genes depicted were predicted transcriptionally differentially expressed (Supplementary File 12) with differential gene expression for husk (red) and IST (blue) illustrated. Gene symbols and B73 AGPv4 gene IDs for the TLP and AL genes at the TF level 2 are noted.

Divergent expression patterns of duplicated genes might be explained by emergent variation in *cis*-regulatory sequences. A promoter analysis was performed to identify potential regulatory elements that may drive tissue-specific expression of these genes within the same gene family. The promoter analysis revealed a 10% or more difference between IST and husk tissue in WRKY and bHLH binding factors in the promoters of the TLP and AL gene families. While WRKY and bHLH factors were predicted to act on TLP genes, only bHLH factors were predicted for AL genes (Fig. 3; Supplementary File 12). This suggests that the activation of TLP and AL genes may be related to the activity of distinct types of TFs in these 2 tissues.

Because the TLP and AL gene families themselves include predicted TFs, differential expression of genes within these families might have led to altered transcriptional activity at other loci. To determine the impact of tissue-specific activation of each distinct family member, regulatory targets of each TLP and AL TF were predicted with the Maize Tissue GRN (https://www.bio.fsu.edu/mcginnislab/mgrn/; Huang *et al.* 2018). The predicted regulatory targets for each AL and TLP family member were compared to the genes identified as differentially expressed and undergoing transcriptional changes via iRNA-seq analysis (Table 7; Supplementary File 12). For all 5 of the TFs analyzed, more than half (∼64%–76%) of the genes that were predicted targets of one of these TFs were transcriptionally differentially expressed rather than post-transcriptionally differentially expressed (Table 7). The 2 AL TFs that were transcriptionally differentially expressed in IST were found to share 36 transcriptionally and 14 post-transcriptionally differentially expressed regulatory targets. This suggested that a hierarchical regulatory network consisting of multiple tiers of TFs may be interacting to create tissue-specific transcription patterns, and the predicted regulatory and coexpression patterns between these genes were used to model a potential GRN for the TLP and AL gene families in husk and IST (Fig. 3).

**Table 7.** Transcriptional activity of TLP and AL genes regulatory targets in husk and IST.

Gene symbol	Tissue^*a*^	Gene regulatory targets			
		Transcriptionally differentially expressed^*b*^	Post-transcriptionally differentially expressed^*c*^	Total number of differentially expressed targets	Total number of targets identified by maize tissue GRN
tubtf5	Husk	230 (71.2%)	93 (28.8%)	323	3,207
tubtf8	IST	847 (72.9%)	315 (27.1%)	1,162	5,874
alf1	Husk	50 (64.1%)	28 (35.9%)	78	996
alf4	IST	263 (72.9%)	98 (27.1%)	361	2,963
alf18	IST	359 (75.7%)	115 (24.3%)	474	2,429

a The tissue identity of the transcriptionally differentially expressed gene according to iRNA-seq analysis.

b The percent of gene regulatory targets that are transcriptionally differentially expressed in relation to the total number of targets differentially expressed is in parentheses (Supplementary File 12).

c The percent of gene regulatory targets with multiple exons post-transcriptionally differentially expressed in relation to the total number of targets differentially expressed is in parentheses (Supplementary File 12).

To predict the biological implications of transcriptional regulation by these TFs, a GO analysis was performed for biological function with the TLP and AL TF regulatory targets using agriGO v2.0 (Tian *et al.* 2017). One of the most prevalent GO terms for the tubtf5 regulatory targets was “response to stimulus” (GO: 0050896; FDR = 8.10E-06; Supplementary File 13), which includes a variety of stimuli including external, abiotic, stress, endogenous, and biotic. Tubtf8 regulatory targets only illustrated “response to abiotic stimulus” (GO:0009628; FDR = 0.0028; Supplementary File 13). In addition, tubtf8 regulatory targets included numerous GO terms involved in developmental processes, like “anatomical structure morphogenesis” (GO:0009653; FDR = 1.40E-09; Supplementary File 13), “post-embryonic development” (GO:0009791; FDR = 1.70E-11; Supplementary File 13), and “flower development” (GO:0009908; FDR = 1.60E-13; Supplementary File 13). The most prevalent GO term for the alf4 regulatory targets was the cellular process “photosynthesis” (GO:0015979; FDR = 9.00E-16; Supplementary File 13). Like the tubtf8 regulatory targets, the alf18 regulatory targets had GO terms associated with developmental processes, like “anatomical structure morphogenesis” (GO:0009653; FDR = 7.80E-05; Supplementary File 13), “post-embryonic development” (GO:0009791; FDR = 5.10E-07; Supplementary File 13), and “flower development” (GO:0009908; FDR = 9.30E-09; Supplementary File 13). No significant GO terms were found for the alf1 regulatory targets (Supplementary File 13). These functions suggest that the TLP TF regulatory targets may be involved in a stimulus or stress response pathway. Interestingly, there were numerous GO terms found to be involved in developmental processes for both tubtf8 and alf18 regulatory targets, which are undergoing active transcription in IST. These regulatory targets may play a role in an IST-tissue-specific developmental response.

## Discussion

iRNA-seq analysis has been used previously to predict genome-wide transcriptional activity in various model systems, including human (Schmidt *et al.* 2015; Kolovos *et al.* 2016; Nacht *et al.* 2016; Madsen *et al.* 2018; Zirkel *et al.* 2018), mouse (Marcher *et al.* 2015; Siersbæk *et al.* 2017; Sustarsic *et al.* 2018), rat (Schmidt *et al.* 2016), and rice (Zhang *et al.* 2018) to address a variety of experimental questions. This study demonstrates that iRNA-seq analysis is an effective way to predict the relative contribution of transcriptional changes to gene expression in maize. Using iRNA-seq analysis, different levels of regulation of gene expression were predicted in IST and husk tissue, leading to insights into possible gene regulatory pathways at the transcriptional and post-transcriptional level. Genes that were identified as being transcriptionally differentially expressed correlated strongly with transcriptional regulatory mechanisms involving TFs and enhancer sequences, which supports the predictions of the iRNA-seq approach. IST and husk tissue are 2 developmentally important tissues in maize, so any insight into the regulation of gene expression in these tissues will increase our understanding of tissue function and tissue differentiation.

The relationship between transcriptional activity and genome duplication was also explored. The modern maize genome is characterized by several genome duplication events, including a relatively recent duplication that is thought to have resulted in 2 distinct subgenomes with characteristic expression levels: Maize1 and Maize2 (Schnable *et al.* 2011; Schnable and Freeling 2011). By comparing subgenome identity to genes exhibiting transcriptional activity, transcriptional activity was found to occur more frequently in the Maize1 subgenome, which supports the previous findings about the subgenomes.

Since genome duplication followed by divergence can create the potential for functional specification between genes with highly similar sequences, we investigated the TLP and AL gene families found in IST and husk tissue for potential expression patterns and regulatory mechanisms. The TLP gene family can be found in all eukaryotes (Liu 2008) and contain a highly conserved tubby domain. In *Arabidopsis*, molecular analysis of the TLP genes found that the TLP genes participate in ABA signaling, a stress response pathway (Lai *et al.* 2004). Another study in maize, also illustrated that the TLP gene family functions in some stress responses, like ABA, temperature, and PEG stress (Yulong *et al.* 2016). This is consistent with the predicted functions of the TLP TF regulatory targets within the gene regulatory model built in this analysis (Fig. 3, Supplementary File 13). The AL gene family is a TF family and has also been shown to play a role in plant abiotic stress responses (Zhou *et al.* 2017). Interestingly, the 2 AL genes found in IST were previously identified as the same subfamily (Zhou *et al.* 2017), which could illustrate some tissue-specificity amongst the gene subfamilies. While other studies examined expression of the TLP and AL genes, the expression analysis did not include IST and husk tissue (Yulong *et al.* 2016; Zhou *et al.* 2017). However, similar tissues were available for comparison. Tubtf5 was found to be expressed in leaves, which is similar to husk tissue, and tubtf8 was found to be expressed in immature leaves, stem, and SAM, which are developing tissues like IST (Yulong *et al.* 2016). Alf1 was found to be expressed in leaf tissue, which is like husk tissue, alf4 was found to be expressed in endosperm and alf18 was found to be expressed in stem and SAM, all of which are developing tissues like IST (Zhou *et al.* 2017). The model for a tissue-specific regulatory network created in this analysis, may therefore, also be relevant to plant stress responses.

In conclusion, iRNA-seq analysis can be used to determine transcriptional activity and changes. Identifying genes transcriptionally differentially expressed has allowed for greater insights into gene expression as well as the prediction of possible GRNs. Additionally, the approach and various tools used in this study could be used to study other pathways, mutants, or tissues in the future.

## Data availability

iRNA-seq analysis pipeline can be found on GitHub (https://github.com/lmschulte/iRNA-seq). This pipeline includes the following plant genomes: maize [AGPv3; AGPv4_ctg (contigs included); AGPv4 (no contigs)] and *Arabidopsis thaliana* (TAIR10). Instructions on how to add additional plant genomes is available on the GitHub page (https://github.com/lmschulte/iRNA-seq).

Supplemental material is available at *G3* online.

## Conflicts of interest 

None declared.

## Supplementary Material

jkac086_Figure_S1

jkac086_File_S1

jkac086_File_S2

jkac086_File_S3

jkac086_File_S4

jkac086_File_S5

jkac086_File_S6

jkac086_File_S7

jkac086_File_S8

jkac086_File_S9

jkac086_File_S10

jkac086_File_S11

jkac086_File_S12

jkac086_File_S13

jkac086_File_S14
