# Supplementary material for: Using iRNA-seq analysis to predict gene expression regulatory level and activity in Zea mays tissues
Source: G3 (Bethesda). 2022 Apr 11;12(6):jkac086. doi: 10.1093/g3journal/jkac086 (PMC9157096; doi:10.1093/g3journal/jkac086)
Supplement: jkac086_Figure_S1 [file jkac086_figure_s1.pdf]

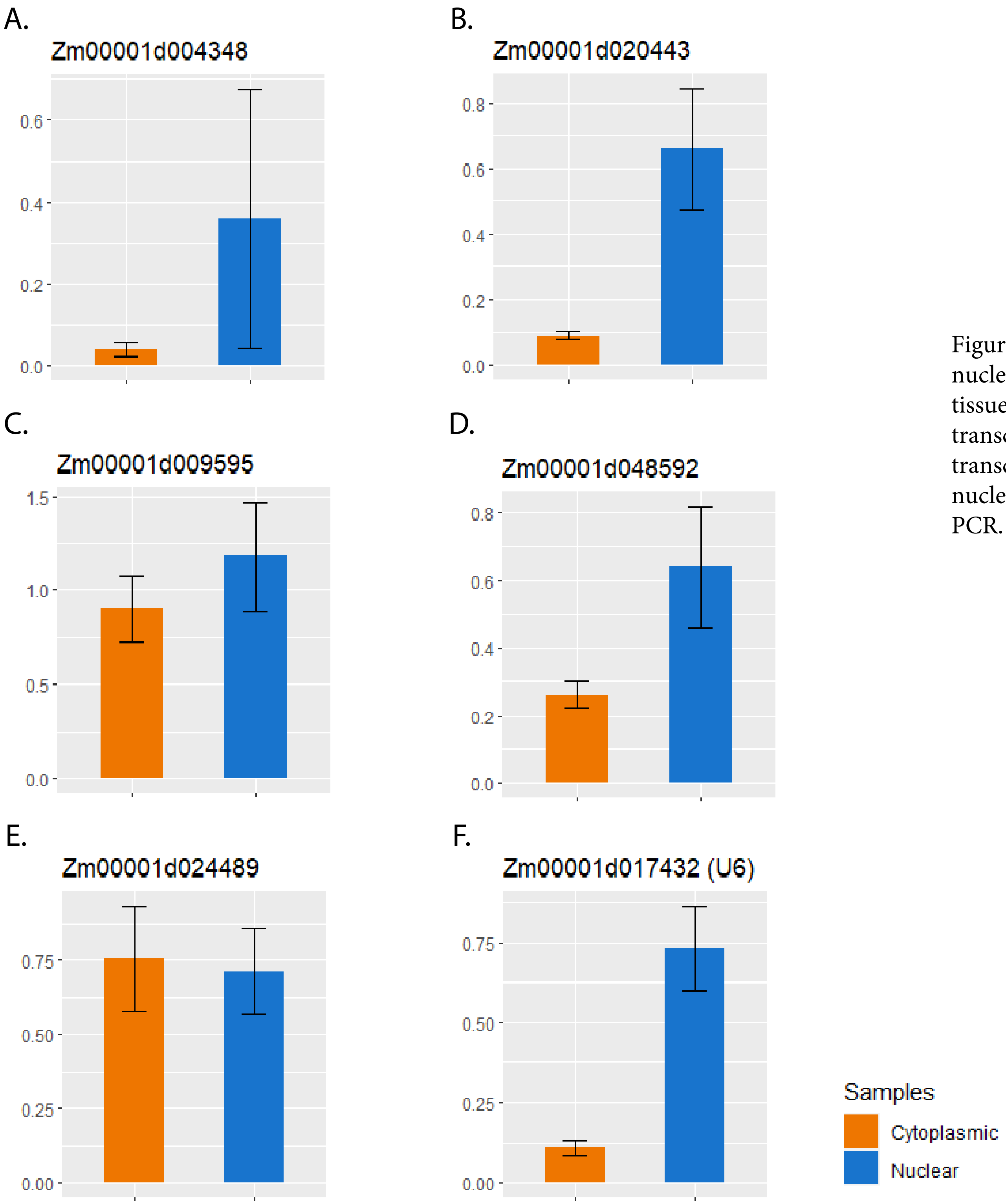

Figure S1 . Differential abundance of mRNAs in nuclear or cytoplasmic fraction of inner stem tissue for genes predicted to be actively transcribed in inner stem (A-C), post transcriptionally regulated in husk (D-E), and a nuclear RNA control (F), as detected by qRT-PCR.
